# Control Strategy Design of a Microblood Pump Based on Heart-Rate Feedback

**DOI:** 10.3390/mi13030358

**Published:** 2022-02-24

**Authors:** Teng Jing, Tianye Xin, Fangqun Wang, Zhihao Zhang, Ling Zhou

**Affiliations:** National Research Center of Pumps, Jiangsu University, Zhenjiang 212013, China; jt-1981@163.com (T.J.); tianye_xin0731@163.com (T.X.); w_fq@163.com (F.W.); zhangzhihao130650@163.com (Z.Z.)

**Keywords:** stroke volume, heart-rate feedback, variable speed, rotary heart pump, hemodynamics

## Abstract

Based on the nonlinear relationship between heart rate and stroke volume, a flow model of left ventricular circulation was improved, and a variable-speed blood-pump control strategy based on heart-rate feedback was proposed. The control strategy was implemented on a system combining the rotary blood pump and blood circulation models of heart failure. The aortic flow of a healthy heart at different heart rates was the desired control goal. Changes in heart rate were monitored and pump speed was adjusted so that the output flow and aortic pressure of the system would match a normal heart in real time to achieve the best auxiliary state. After simulation with MATLAB, the cardiac output satisfied the ideal perfusion requirements at different heart rates, and aortic pressure demonstrated lifting and had good pulsatile performance when a variable-speed blood pump was used. The coupled model reflected the relationship between hemodynamic parameters at different heart rates with the use of the variable-speed blood pump, providing a theoretical basis for the blood-pump-assisted treatment of heart failure and the design of physiological control strategies.

## 1. Introduction

Over the last decades, medical researchers have placed increased emphasis on the development of left ventricular assist devices (LVADs), which can enhance or substitute the function of the natural heart and improve the blood perfusion levels of patients. In clinical practice, LVADs are usually implanted for the targeted treatment of end-stage congestive heart failure or short-term support for patients waiting for heart transplantation [1,2,3,4,5].

To date, the constant-speed blood pump is widely used in the clinical application of LVADs. This pump provides stable blood flow that differs from physiological flow because it lacks pulsatility [6,7,8]. A reduction in pulsatility causes persistent nonphysiological pressure at the aortic root; thus, aortic root dilatation and valve fusion occur, which lead to some complications such as platelet dysfunction, endothelial dysfunction, and gastrointestinal bleeding [9,10,11,12]. In addition, the speed range of the constant-speed blood pump is narrow. An extremely slow pump speed may result in insufficient supply when blood regurgitates back from the aorta. Conversely, an extremely fast pump speed leads to serious suction, which causes myocardial damage and ventricle collapse [13,14,15,16].

To provide patients with as normal a lifestyle as possible, many control algorithms were proposed. For instance, some simulations allow the imposition of a pulsatile operation mode by adding the sinusoidal modulation of rotational speed [17,18,19,20]. However, resulting pressure and flow waveforms did not accord with the characteristics of the true pulsatile flow of the heart. Chen et al. [21] used a feedback mechanism that adopted minimum pump-flow gradients in real time to control pump speed. However, the objective of this controller was to prevent the occurrence of suction, and whether the optimal flow perfusion level of patients was satisfied was not considered. According to the Frank Starling mechanism, some developed pump-flow adaptive controllers adjust pump flow in accordance with changes in left ventricular preload [22,23,24,25]. Although these kinds of controllers have better performance than constant-speed operation in vivo tests [26,27], pump speed remains constant in the local range when preload remains stable in a certain period of time. Petrou et al. [28] developed a multi-objective physiological control system that was dependent on pump inlet pressure (PIP) and implemented signal processing algorithms to extract features from PIP that can meet various objectives; however, the control method relied on the development of a blood-pressure sensor with good blood compatibility. Leao et al. [29] proposed a multi-objective physiological control system based on fuzzy logic to maintain the mean arterial pressure and cardiac output at the physiological level.

Based on the above studies, the purpose of this study was to develop a control strategy that coincides with physiological changes in the human body. The control strategy would not use additional sensors for hemodynamic parameters. The aortic flow of a healthy heart at different heart rates was the desired goal, and the rotation speed of the blood pump was adjusted synchronously according to heartbeat cycle so that the output flow and aortic pressure of the combined blood-pump system can match a normal heart in real time to achieve the best auxiliary state.

## 2. Methods

Heart rate was used as a feedback variable in the regulation of variable-speed blood-pump rotation so that the blood pump would adapt to physiological changes. Given that the heart rate fluctuates during slight physical activity or emotional changes, it can reflect the blood perfusion demand of a patient to some extent.

### 2.1. Aortic Flow Models at Different Heart Rates

**Figure 1:**
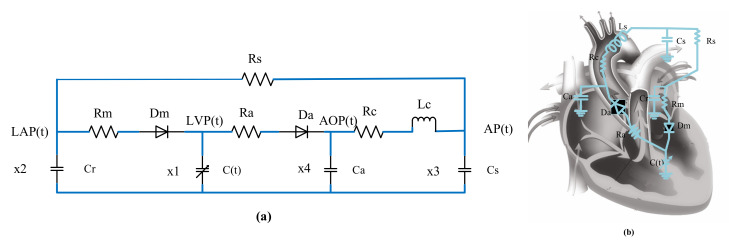
Electric circuit model. (**a**) 0D lumped-parameter circuit model of left ventricular circulation, (**b**) Equivalent circuit of blood circulation.

**Figure 2:**
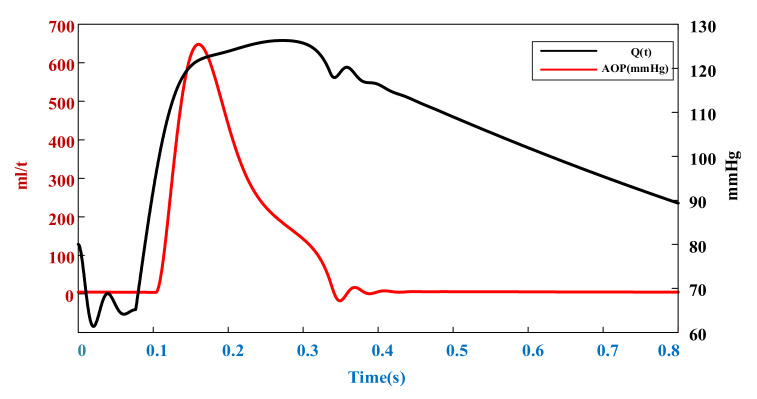
Healthy aortic flow and pressure curve at rest (heart rate = 75 bpm).

According to the similarity between the blood circulatory system and circuit loop, the cardiovascular circulatory system was simulated with a 0D circuit model [30,31,32]. In the design of cardiovascular circulatory system models, a fifth-order lumped-parameter electric circuit model (Figure 1) that can reflect left-heart hemodynamics can be established by neglecting the pulmonary circulatory system and right-heart function, owing to the low impact of the pulmonary circulation and right ventricle on the heart failure environment. By substituting corresponding initial parameters and performing iterative calculation with MATLAB (2021b, MathWorks, Inc., Natick, MA, USA), the aortic flow and pressure curve (Figure 2) of the resting heart rate (75 bpm) under healthy conditions were obtained. Stroke volume at this heart rate can be calculated by integrating time into the cardiac cycle of the flow curve. The parameter values of the 0D lumped-parameter circuit model are shown in Table 1.

The method of noninvasive measurement of stroke volume is usually based on the principle of chest impedance. This type of research is gradually modified from the Nyboer equation:

(1) $$\;\mathrm{SV}=\frac{{\rho L}^{2}}{Z_{0}^{2}}\cdot \Delta Z.$$

Among them, the most famous Kubicek equation [33] is to replace ΔZ in Nyboer equation with ${\left(\mathrm{dZ}/\mathrm{dt}\right)}_{\max}\cdot {\;T}_{s}$, The formula would then be:

(2) $$\;\mathrm{SV}=\frac{{\rho L}^{2}}{Z_{0}^{2}}\cdot {\left(\frac{\mathrm{dZ}}{\mathrm{dt}}\right)}_{\max}\cdot T_{s}.$$

However, the derivation of Nyboer theory assumes that the blood injected into the aorta during left ventricular contraction does not flow into the peripheral blood vessels. This assumption does not correspond to actual physiological conditions. Therefore, an improved model is used in this paper, as shown in Formula (3) [34].

(3) $$\mathrm{SV}=\frac{{K\rho L}^{2}}{Z_{0}^{2}}\cdot {\left(\frac{\mathrm{dZ}}{\mathrm{dt}}\right)}_{\max}\cdot T_{s}$$

where K is the time constant, which is equal to the ratio of the time during the cardiac cycle; diastole (T_d_) and (T_s_) represent contraction time; ρ is the resistivity of blood, which can be regarded as a constant, generally taken as 135 Ω·cm; L is the distance between the detection electrodes; Z_0_ is the basic impedance; and (dz/dt) is the slope of a point on the impedance diagram. The variables ρ, L, Z_0_, $\frac{\mathrm{dz}}{\mathrm{dt}}$, are all variables independent of heart rate and can be represented by $f=\rho {\left(\frac{L}{Z_{0}}\right)}^{2}\frac{\mathrm{dz}}{\mathrm{dt}}$. Stroke volume (5) can be obtained by performing the substitution in the linear regression equation for heart rate (4) [35] and left ventricular ejection time. The results calculated by this model are highly consistent with the regression curves of heart rate and cardiac volume fitted by Gu Kaiyun through experimental data [36], so the accuracy has been verified.

(4) $$T_{s}=0.413-0.0017HR$$

(5) $$SV=f\frac{60{\left(0.413-0.0017HR\right)}^{}}{60-0.413HR+0.0017HR^{2}}$$

**Figure 3:**
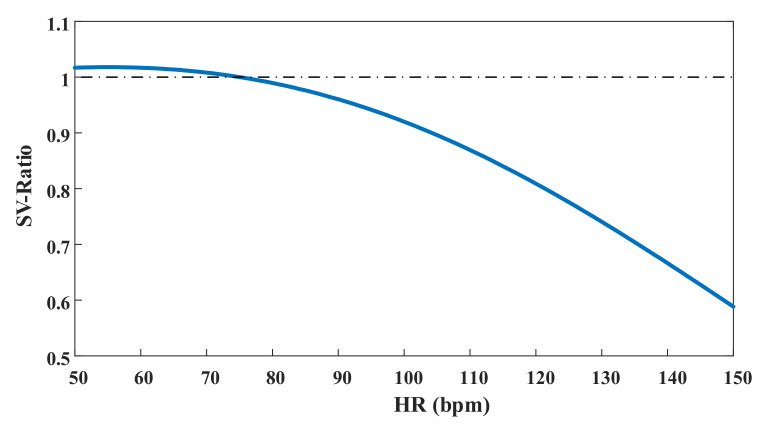
Relative value of SV under different heart rates.

By introducing different heart rates into the above equation and dividing them by stroke volume at a heart rate of 75 bpm, we determined the ratio of stroke volume under each heart rate with the SV at rest, as showed in Figure 3.

The expected stroke volume at different heart rates can be calculated by multiplying the ratio of stroke volume at different heart rates by the stroke volume at a heart rate of 75 bpm. Then, according to the original fifth-order equation for obtaining the value of the aortic flow curve under different heart rates, the correction coefficient K was calculated using MATLAB. Stroke volume was calculated after aortic flow was multiplied by the corresponding K for the integration of time that can be equal to the expected value. Finally, aortic flow curve models with different heart rates can be obtained by multiplying the K value by the corresponding flow value.

### 2.2. Left-Heart-Circulation Coupling Model with Blood-Pump Assistance

**Figure 4:**
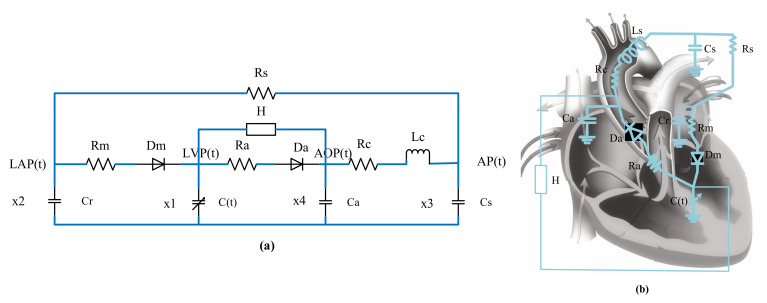
Updated electric circuit model. (**a**) 0D lumped-parameter model of parallel rotating blood pump for left ventricular assist, (**b**) Equivalent circuit of blood circulation.

The ventricular assist device used is a centrifugal blood pump that we developed autonomously. The blood pump belongs to the third-generation rotary blood pump, and the impeller has high working efficiency. Therefore, the rotating speed can be reduced between 2000–6000 rpm, and the flow also reaches 2–8 L/min, which not only is less damaging to the blood, but also can meet the needs of different working conditions in different heart rates. It is the equivalent of a centralized parameter model. A left-heart-circulation coupling model under blood-pump assistance was established by connecting the device in parallel to a centralized parameter model of left-heart circulation. The circuit diagram is provided in Figure 4.

According to the characteristic curve of the centrifugal blood pump and the relationship among the head, flow, and speed, the mathematical model expression of the centrifugal blood pump was obtained [37].

(6) $$H=p_{0}-p_{i}=\beta_{0}*Q+\beta_{1}*\frac{dQ}{dt}+\beta_{2}*\omega^{2}$$

where H is the head of the pump; $P_{0}$, ${\;P}_{i}$ are the pump’s out pressure and inlet pressure, respectively; Q is the pump flow; $\frac{\mathrm{dQ}}{\mathrm{dt}}$ is the rate of change of pump flow; ω is the pump speed; $\beta_{0}$ is the pump resistance coefficient with a value of −0.391; $\beta_{1}$ is the pump inertia coefficient with a value of −0.00183; and $\beta_{2}\;$ is the   correlation constant of the pump, with a value of 1.147 ×$\;10^{-5}$.

The parameter settings and values in the model are shown in Table 2, where $E_{\max}$ values of 1.05, 0.835, 0.713, and 0.63 mmHg/mL can be used in the simulation of grades I–IV heart failure, respectively. ($E_{\max}$ is related to the end-systolic pressure–volume relationship).

**Figure 5:**
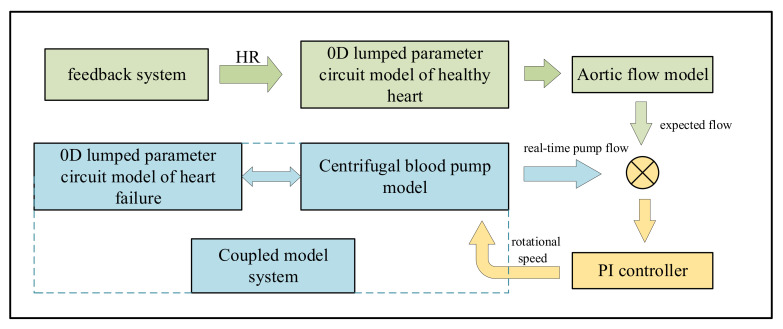
The flow chart of feedback control.

**Figure 6:**
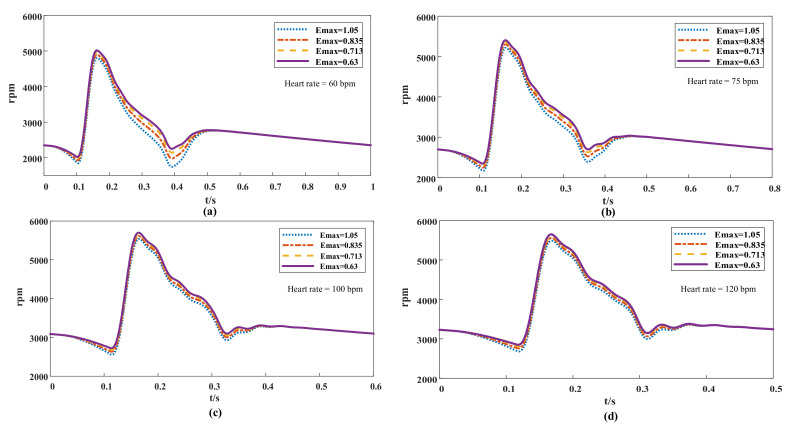
Variable-speed image under different $E_{\max}$ in a cardiac cycle at different heart rates. (**a**) heart rate = 60 bpm, (**b**) heart rate = 75 bpm, (**c**) heart rate = 100 bpm, (**d**) heart rate = 120 bpm.

Figure 5 shows the lumped-parameter model of the parallel connection of the centrifugal blood pump and left ventricular circulation system. According to Kirchhoff’s law, the state equation of the equivalent circuit of the model can be listed, as shown in Formula 5.

(7) $$\left\{\begin{array}{c} \frac{dx_{1}\left(t\right)}{dt}=\left[\frac{r\left(x_{2}\left(t\right)-x_{1}\left(t\right)\right)}{Rm}-x_{6}\left(t\right)-\frac{r\left(x_{1}\left(t\right)-x_{4}\left(t\right)\right)}{Ra}-x_{1}\left(t\right)*\frac{dC(t)}{dt}\right]/C\left(t\right)\\ \frac{dx_{2}\left(t\right)}{dt}=\left[\frac{x_{3}\left(t\right)-x_{2}\left(t\right)}{Rs}-\frac{r\left(x_{2}\left(t\right)-x_{1}\left(t\right)\right)}{Rm}\right]/Cr\\ \frac{dx_{3}\left(t\right)}{dt}=\left[x_{5}\left(t\right)-\frac{x_{3}\left(t\right)-x_{2}\left(t\right)}{Rs}\right]/CS\\ \frac{dx_{4}\left(t\right)}{dt}=\left[\frac{r\left(x_{1}\left(t\right)-x_{4}\left(t\right)\right)}{Ra}-x_{5}\left(t\right)+x_{6}\left(t\right)\right]/Ca\\ \frac{dx_{5}\left(t\right)}{dt}=\left[x_{4}\left(t\right)-x_{3}\left(t\right)-Rc*x_{5}\left(t\right)\right]/Ls\\ \frac{dx_{6}\left(t\right)}{dt}=\left[x_{4}\left(t\right)-x_{1}\left(t\right)-\beta_{0}*x_{6}\left(t\right)-\beta_{2}*\omega^{2}\right]/\beta_{1} \end{array}\right.$$

where r (ξ) is a slope function, which can be used to indicate the state of the mitral and aortic valves (Figure 6):

(8) $$r\left(\xi \right)=\left\{\begin{array}{c} \xi ,if\;\xi \geq 0\\ 0,if\;\xi <0 \end{array}\right.$$

According to the above equation, different $E_{\max}$ values were selected and substituted into corresponding initial parameters. The flow and pressure curves of different heart-failure grades were obtained using MATLAB.

### 2.3. Feedback Control of Heart Rate

According to the heart rate detected in real time, the aortic flow of a healthy heart under different heart rates was calculated, and the flow was regarded as the expected flow in pump control. When PI control was adopted, the output flow of the coupling system with left-heart failure circulation assisted by blood pump was consistent with a healthy heart, and the required pump speed was determined, as show in Figure 5.

## 3. Results

MATLAB was used in simulating the coupled-model system and solving the corresponding pump speed. The results were used in evaluating the effects of the variable-speed control strategy based on heart-rate detection (Figure 6). After the heart rate of the model was changed, the hemodynamic parameters of the blood circulation model at variable speeds were tested. The fluctuation range of flow and blood pressure in the left ventricular circulation system was inspected after it was pump-assisted, and whether it met the physiological requirements under the health condition was determined. To reflect the operational effect of the control strategy, a coupling model that can reflect the blood pressure and flow after constant-speed (3000 rpm) blood-pump assistance was established in the simulation experiment.

Given that fluctuations in heart rate can lead to changes in the physiological parameters of patients at any time, representative heart rates of 60, 75, 100, and 120 bpm were selected for analysis and comparison (because the heart rates of normal people in quiet state is generally 60–100 bpm, the average heart rate is 75 bpm, and 120 bpm can represent the state of slight exercise or tachycardia).

### 3.1. Flow Simulation of Different Controllers

**Figure 7:**
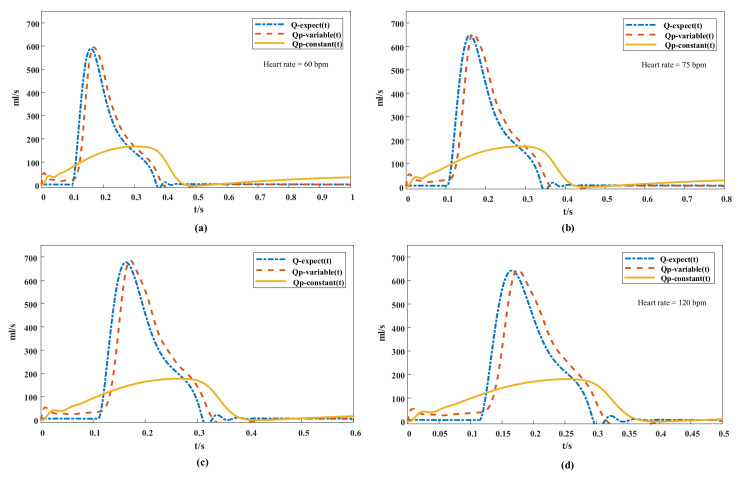
Comparison of flow curve at different heart rates: Q-expect (t) is the expected quantity of flow, QP -variable (T) is the variable-speed pump flow, QP-constant (T) is the constant-speed pump flow. (**a**) heart rate = 60 bpm, (**b**) heart rate = 75 bpm, (**c**) heart rate = 100 bpm, (**d**) heart rate = 120 bpm.

The simulation of grade IV ($E_{\max}\;$= 0.63) heart failure for the flow curve is shown in Figure 7. The expected aortic flow curve was depicted, and the flows of variable-speed and constant-speed pumps in a cardiac cycle at different heart rates were compared. Under the PI control of variable-speed strategy, the pump flow curve tended to be consistent with the expected flow curve; and the phase lag was only 0.02 s, and more synchronized after the ejection of the heart than that of the constant-speed blood pump.

**Figure 8:**
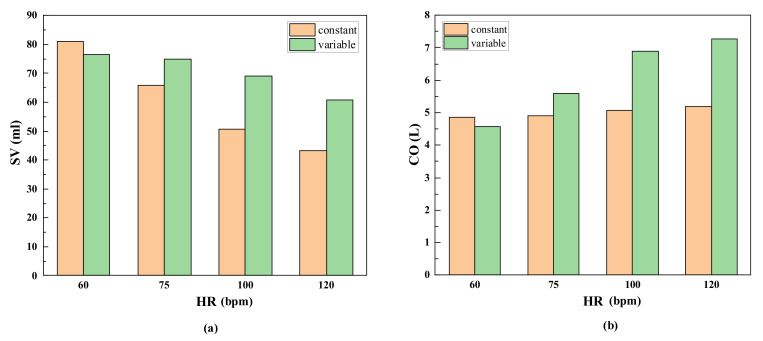
Comparison of flow output under two control strategies. (**a**) the stroke volume at different heart rates, (**b**) the cardiac output after pump assistance.

The pump flow curve of each cardiac cycle was integrated using MATLAB, and the stroke volume at different heart rates (Figure 8a) was calculated using the two control strategies. After the first two cardiac cycles, stroke volume was multiplied by the corresponding heart rate for the calculation of the cardiac output after pump assistance (Figure 8b). For the variable-speed pump designed in this study, the stroke volumes of each pulse were approximately 76.4, 75, 69, and 60 mL when the heart rates were 60, 75, 100, and 120 bpm, respectively, meeting the preset perfusion requirements [19,38]. For the constant-speed pump, although the cardiac output slightly increased with increasing heart rate, the difference between the output and expected perfusion at the corresponding heart rate increased.

### 3.2. Pressure Simulation of Different Controllers

**Figure 9:**
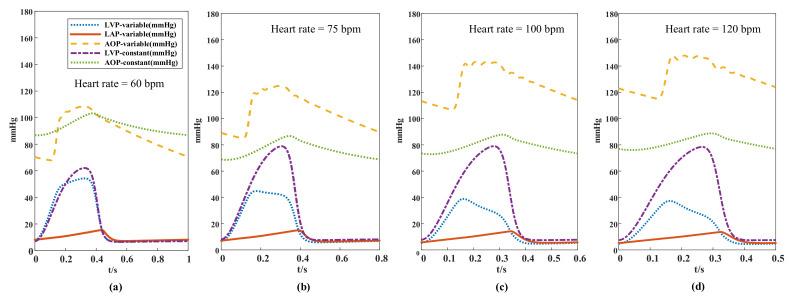
The pressure waveforms of different heart rates in the two auxiliary strategies; LVP-variable: left ventricular pressure with variable-speed assist; LAP-variable; left atrial pressure with variable-speed assist; AOP-variable: aortic pressure with variable-speed assist; LVP-constant with variable-speed assist: Left ventricular pressure with constant-speed assistance; LAP-constant: left atrial pressure with constant-speed assistance. (**a**) heart rate = 60 bpm, (**b**) heart rate = 75 bpm, (**c**) heart rate = 100 bpm, (**d**) heart rate = 120 bpm.

The simulation of grade IV ($E_{\max}$ = 0.63) heart failure for the pressure waveform is shown in Figure 9, where changes in pressure in the blood circulation system can be seen at different heart rates and under two control strategies. At an accelerated heart rate, aortic pressure increased gradually and fluctuated between 69–108, 84–124, 106–143, and 116–148 mmHg when the heart rates were 60, 75, 100, and 120 bpm, respectively. The range of increase in aortic pressure gradually decreased with increasing heart rate, and the pressure fluctuation conformed to the range in healthy people and showed physiological characteristics. By contrast, under constant speed operation, the aortic pressure and left ventricular pressure were between 88–96 mmHg and between 73–77 mmHg, respectively, and did not change with heart rate when the constant-speed pump system was used. No significant difference in left atrial pressure between the two strategies was observed, and the peak pressure difference was slightly higher when the variable-speed pump was used and consistently within a range of 5–15 mmHg.

**Figure 10:**
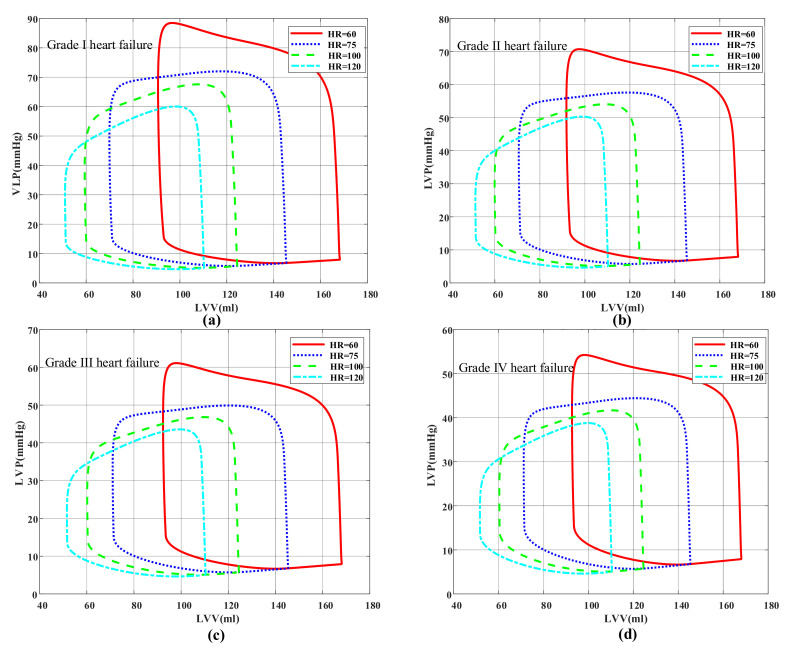
Pressure–volume loop. (**a**) Grade I heart failure ($E_{\max}$ = 1.05), (**b**) Grade II heart failure ($E_{\max}\;$= 0.83), (**c**) Grade III heart failure ($E_{\max}\;$= 0.713), (**d**) Grade IV heart failure ($E_{\max}$ = 0.63).

The effect of the variable-speed control strategy was evaluated. Different conditions of heart failure were added to the model, and then the physiological state of pump assistance under different heart rates and degrees of myocardial elasticity were investigated. The pressure–volume loop (Figure 10) is used in evaluating cardiac function [39,40]. Although the loop had no definite time, it was drawn according to the pressure and volume at each time point of the cardiac cycle, which can directly reflect the relationship between ventricular pressure and volume in different periods, and then the physiological state was analyzed when a pump was used. According to the different grades of heart failure, the effects of heart-rate changes on hemodynamics in different physiological states were studied. The volume in the end period of ventricular diastole increased with decreasing heart rate, resulting in a high preload. Thus, the corresponding stroke volume increased (the stroke volume is equal to the width of the closed loop). This result was consistent with the Frank–Starring curve [41]. At the same heart rate, the elastic condition of the heart worsened as the $E_{\max}$ value decreased, and thus the blood pump increased the speed to improve the auxiliary ratio (Figure 6) and subsequently lowered pressure at the end period of ventricular systole. The results showed that the variable-speed control strategy can provide a healthy pressure pulsation environment for the coupled model of left ventricular circulation, and the control system can be adjusted through real-time heart-rate feedback to achieve the ideal cardiac output.

## 4. Discussion

The outcomes of the variable-speed control strategy and constant-speed blood pump operation were compared, and physiological analysis was performed on the flow and pressure simulation results generated by the model. We used the results in exploring the interactions of different control types of left ventricular assist devices with the cardiovascular system and discussing the advantages and disadvantages of the variable-speed control strategy.

In the aortic flow simulation of the natural heart in Figure 7, when the heart rate increased from 60 bpm to 120 bpm, the time proportion of the systolic period in the cardiac cycle increased from 28.5% to 37.6% despite the time of the cardiac cycle being shortened. Thus, at increased heart rate, the diastolic time of the heart was shortened more significantly than the systolic time; because the variable-speed blood pump was adjusted under PI control, the proportion of low-speed operation and high-speed operation was reduced under the same cardiac cycle. This result was in line with the blood circulation system physiology of healthy conditions [42] and has important significance for explaining changes in stroke volume.

Stroke volume is the difference between end-diastolic volume and end-systolic volume, so it is essential to the analysis of changes in diastolic and stroke volumes (Figure 8). When the heart rate increased from 75 bpm to 100 bpm, most of the venous-return blood flowed into the ventricle during the rapid filling period, even though the ventricular filling time decreased. Hence, the end-diastolic volume and stroke output did not decrease significantly. However, the cardiac output increased significantly in ejection time. When the heart rate increased from 100 bpm to 120 bpm, the period of ventricular diastole was significantly shortened, resulting in insufficient ventricular filling time and decreased venous-return blood volume, which led to stroke volume decreases in the end diastolic. However, owing to an increase in ejection frequency, the total cardiac output increased relative to the resting state. When the heart rate dropped from 75 bpm to 60 bpm, although the diastolic period was prolonged, the left ventricular filling volume was close to the maximum, and thus the increase in stroke volume was extremely limited, so the cardiac output reduced due to the slow heart rate. Cardiac output was reduced relative to that at the resting state. According to the literature, the cardiac outputs of healthy adults are approximately 5 L/min, and the output flow of the blood pump was generally 4–5 L/min [43]. In the blood pump running at constant speed, the blood transfusion volume reached 4.85–5.18 L/min at 3000 rpm (Figure 8b). Although the total output of the pump increased with heart rate [44,45], the increase in range was narrower than that in the variable-speed control pump. In addition, although the cardiac output of the constant-speed blood pump reached a rational perfusion volume by increasing pump flow during the diastolic period, this will greatly increase the probability of suction. [46] (Figure 7).

Regarding the pressure waveforms (Figure 9) of different heart rates in the two control strategies, the aortic pressure ranges in pump-speed controllers decreased with increasing heart rate. For instance, at a heart rate of 60 bpm, the aortic pressure difference in the variable-speed and constant-speed pumps were 40 and 16 mmHg, respectively, indicating that the pulsatility of aortic pressure controlled by the variable-speed pump was stronger than that controlled by the constant-speed pump and could reduce the risk of clinical diseases caused by decreased pulsatility.

The pressure ranges of the left ventricular were 54–57 mmHg for the variable-speed control and 62–67 mmHg for the constant-speed control. The left ventricular pressure was consistently lower than the pressure in the aorta, indicating that the aortic valve remained closed all the time. Given that the speed of the blood pump changed according to the variations in the cardiac cycle to work synchronously with the heart, it unloaded the left ventricular function in the ejection phase, thereby considerably reducing left ventricular pressure. The left ventricular pressure was lower when the variable-speed pump was used because it worked more in the ejection phase, and the unloading effect on the ventricular was more obvious. No substantial difference in left atrial pressure was observed between the auxiliary strategies, because left atrium pressure was mainly affected by the volume of venous blood returned.

There are also some contents worth discussing regarding the image with the speed of blood pump. Figure 6 shows that the peak value of the pump speed did not decrease at increased heart rate and reduced stroke volume, but instead presented an opposite change. The reason was that the flow of the pump was not only affected by pump speed but was also related to the pump head. Figure 9 shows that the difference between the aortic pressure and left ventricular pressure was small when the heart rate was low, indicating that the head of the pump was low at this time. Given that the pump head was inversely proportional to the flow rate, the blood pump did not need a high speed to produce an abundant flow rate. When the heart rate increased, the head of the pump increased, along with the pressure difference between the aorta and left ventricle. Therefore, the blood pump increased the speed to reduce the flow loss caused by the rise of the pump head.

In conclusion, (1) compared with constant-speed control, the variable-speed control proposed in this study can adjust pump-flow output according to heart-rate feedback to meet the optimal blood perfusion level; (2) the pulsatile pressure of a heart with heart failure was increased to maintain the aortic pressure in the normal range; (3) the output mode of pump flow was highly consistent with the original healthy heart, which reduced the risk of suction and backflow. Compared with the other variable-speed control strategies, the proposed control strategy can calculate the ideal perfusion flow according to heart-rate feedback. In contrast to most physiological controllers, the proposed strategy does not need additional sensors for decision making, lowers the risk of thrombosis, and does not reduce the reliability of a system. Computational simulations showed that it can generate adequate blood flow and pressure and does not possess the disadvantages of insufficient accuracy caused by equipped sensors [47,48]; and (4) the control strategy proposed in this paper is suitable for all kinds of rotary blood pumps, as long as the operating conditions of the blood pump under different requirements are known, and its operating range meets the requirements of variable-speed control.

This study contains some limitations, which need to be analyzed and addressed in the future: (1) Given that heart failure is accompanied by weakened myocardial contractility, the metabolic acceleration of heart rate is often observed at decreased cardiac output [49]. Therefore, the cardiac output after assistance may be higher with theoretical value. This approach normalizes the reserve of systolic and diastolic, and the heart rate returns to a healthy state. However, the overall cardiac output may become higher than the expected value. Therefore, the validity of the simulation results of the control model needs to be further verified by in vitro experiments. (2) In the simulation of the control model, the aortic valve remained closed because the left ventricular pressure was lower than the aortic pressure. In this case, it may cause adhesion between aortic leaflets and damage the aortic valve [50]. An aortic-valve control module can be added to adjust pump speed within a certain period of time to promote intermittent aortic-valve opening. (3) The peripheral resistance of cardiopulmonary bypass changes with the exercise state, but in this paper, the constant value of resistance is used to represent the peripheral resistance, and this factor is not considered. However, this is not the focus of this paper. If the model of the change in blood-resistance heart rate is obtained in the latter study, it can be directly added into the control strategy to obtain more accurate control results. (4) In this paper, the pump speed generated by the control system changed in real time. It increased synchronously in the ejection phase to meet the perfusion requirements of the patients. In the diastolic phase, the pump speed was reduced until the aortic pressure alone would be maintained. When pump flow was almost zero, it was highly consistent with the beating state of the natural heart. However, a sudden change in speed may cause potential blood damage and should thus be analyzed using in vitro experiments. In addition, achieving sensitive speed change in impeller blood pumps may be a problem in experiments. Nevertheless, advanced equipment such as HeartMate 3 implements algorithms for rapidly changing pump speed [28].

## 5. Conclusions

A 0D lumped-parameter model coupled with the left ventricular assist device model and the circuit model of the blood circulation system was established, and the pump speed curve of the ventricular assist device at variable optimal speed was analyzed under different heart failure and heart rates. Then, according to the nonlinear relationship between heart rate and stroke volume, a new variable-speed control strategy was established, which used heart rate as a feedback signal, and compared with the physiological parameters of constant-speed control. The results showed that the coupling models with different degrees of heart failure can achieve ideal stroke volume after the addition of pump-speed controllers, and the pulsatility of aortic pressure is better than that of a constant-speed pump controller.

## Figures and Tables

**Table 1:** Parameters are used in 0D lumped-parameter circuit model.

Parameter	Value	Physiological Meaning
Rm	0.0050 mmHg·s/mL	Mitral valve resistance
Ra	0.0010 mmHg·s/mL	Aortic valve resistance
Rc	0.0398 mmHg·s/mL	Aortic resistance
Rs	1.0000 mmHg·s/mL	Systemic vascular resistance
Cr	4.4000 mmHg·s/mL	Left atrial compliance
C(t)	Time-varying	Left ventricular compliance
Ca	0.0800 mmHg·s/mL	Aortic compliance
Cs	1.3300 mmHg·s/mL	Peripheral vascular compliance
Ls	$0.0005\;\text{mmHg}\cdot s^{2}$/mL	Aortic blood inertia
Dm		Mitral Valve
Da		Aortic Valve

**Table 2:** State variables of the left ventricular circulation system.

Variable	Significance	Definition	Initial Value
x1(t)	Left ventricular pressure	LVP(t)	7.6 mmHg
x2(t)	Left atrial pressure	LAP(t)	7.6 mmHg
x3(t)	Arterial pressure	AP(t)	67 mmHg
x4(t)	Aortic pressure	AOP(t)	80 mmHg
x5(t)	Aortic flow	Q(t)	0 mL/s
x6(t)	Pump flow	Qp(t)	0 mL/s
